# A prognostic model based on gene expression parameters predicts a better response to bortezomib-containing immunochemotherapy in diffuse large B-cell lymphoma

**DOI:** 10.3389/fonc.2023.1157646

**Published:** 2023-04-28

**Authors:** Adrián Mosquera Orgueira, Jose Ángel Díaz Arías, Rocio Serrano Martín, Victor Portela Piñeiro, Miguel Cid López, Andrés Peleteiro Raíndo, Laura Bao Pérez, Marta Sonia González Pérez, Manuel Mateo Pérez Encinas, Máximo Francisco Fraga Rodríguez, Juan Carlos Vallejo Llamas, José Luis Bello López

**Affiliations:** ^1^ University Hospital of Santiago de Compostela, Servizo Galego de Saúde (SERGAS), Santiago de Compostela, Spain; ^2^ Health Research Institute of Santiago de Compostela, Santiago de Compostela, Spain

**Keywords:** machie learning, DLBCL - diffuse large B cell lymphoma, bortezomib, R-CHOP, lymphoma, genomics, gene expression

## Abstract

Diffuse Large B-cell Lymphoma (DLBCL) is the most common type of aggressive lymphoma. Approximately 60% of fit patients achieve curation with immunochemotherapy, but the remaining patients relapse or have refractory disease, which predicts a short survival. Traditionally, risk stratification in DLBCL has been based on scores that combine clinical variables. Other methodologies have been developed based on the identification of novel molecular features, such as mutational profiles and gene expression signatures. Recently, we developed the LymForest-25 profile, which provides a personalized survival risk prediction based on the integration of transcriptomic and clinical features using an artificial intelligence system. In the present report, we studied the relationship between the molecular variables included in LymForest-25 in the context of the data released by the REMoDL-B trial, which evaluated the addition of bortezomib to the standard treatment (R-CHOP) in the upfront setting of DLBCL. For this, we retrained the machine learning model of survival on the group of patients treated with R-CHOP (N=469) and then made survival predictions for those patients treated with bortezomib plus R-CHOP (N=459). According to these results, the RB-CHOP scheme achieved a 30% reduction in the risk of progression or death for the 50% of DLBCL patients at higher molecular risk (p-value 0.03), potentially expanding the effectiveness of this treatment to a wider patient population as compared with other previously defined risk groups.

## Introduction

Diffuse Large B-cell Lymphoma (DLBCL) is the most common type of aggressive lymphoma. Approximately 60% of patients achieve curation with the standard first line treatment, which is based on the combination of an anti-CD20 antibody (rituximab) with chemotherapy (cyclophosphamide, doxorubicin and vincristine) and prednisone (R-CHOP). The remaining patients have either refractory disease or relapse after achieving a remission, and this predicts an adverse prognosis (1). Traditionally, risk stratification has been based on scores that combine the value of different prognostic variables. Examples of these methods are the International Prognostic Index (IPI), the revised IPI (R-IPI), and the National Comprehensive Cancer Network IPI (NCCN-IPI) (2). Nevertheless, the accuracy of these scores is far from optimal, and other strategies are actively being explored based on novel molecular features. Earlier studies based on transcriptomic signatures revealed 3 prognostic groups based on their cell-of-origin (COO) status: activated B-cell–like (ABC), germinal-center B-cell–like (GCB) and unclassified (3). Furthermore, recent research reports proved that high risk lymphomas can also be identified as those which share a gene expression signature with either double & triple-hit DLBCLs or with Burkitt lymphomas (4). These lymphomas have been termed as molecular high-grade (MHG) by the academics (4). Finally, comprehensive classifications of DLBCL based on patterns of somatic mutations also exist, which are also associated with divergent clinical outcomes (5).

A few years ago, we presented a new prognostic tool in DLBCL based on a 102-gene expression profile (6). The data from this profile, when interpreted with machine learning tools, enabled the inference of personalized survival outcomes that were prognostically superior to those of the COO classification. Afterwards, we reproduced this profile in another cohort, and reduced the total number of genes in the signature to 19 variables which were prognostically independently of the IPI-related variables (7). The model was named LymForest-25. Finally, we validated the prognostic value of this signature in the UK population-based Haematological Malignancy Research Network database (8), confirming its superiority with respect to the COO and MHG classifications. Notably, the performance of the predictor continued to be high despite the exclusion of 2 genes which were not represented in the gene expression panel used in that study.

At the same time, a growing interest for improved treatments in DLBCL has emerged, and several trials have evaluated new upfront combinations during the last years. The ROBUST study was a randomized, phase III trial which explored the addition of lenalidomide to R-CHOP (R2-CHOP) vs standard R-CHOP, but failed to provide significant results (9). However, a tendency for an improved progression-free survival (PFS) with R2-CHOP was observed among patients with high risk disease (IPI ≥ 3). More recently, the POLARIX phase III trial evaluated a modified scheme of R-CHOP (pola-R-CHP), in which vincristine was replaced with polatuzumab vedotin, as compared with standard R-CHOP in patients with previously untreated intermediate-risk or high-risk DLBCL (10). A significant benefit in PFS was observed in the pola-R-CHP treatment branch, with a hazard ratio (HR) of 0.73. Notably, exploratory subgroup analysis evidenced that this benefit was more pronounced among patients with IPI ≥ 3 and in those with ABC phenotype. A different therapeutic strategy has been based on the incorporation of the proteasome inhibitor bortezomib into the R-CHOP scheme (RB-CHOP). Preclinical evidence indicated that bortezomib can exert antitumoral activity in B-cell lymphoma cell lines (11). This promoted clinical studies that ended up in the development of the REMoDL-B trial, a randomized phase III trial testing RB-CHOP vs R-CHOP in previously untreated DLBCL patients (12). The results of this trial indicated no evidence for a benefit of RB-CHOP over R-CHOP neither in PFS nor in overall survival (OS). However, exploratory *post-hoc* analysis evidenced a benefit for RB-CHOP in PFS in the MHG group, and a tendency towards a benefit in the ABC group (13).

## Methods

In the present report, we aimed to reproduce the prognostic value of the LymForest gene expression profile in the publicly available data of the REMoDL-B trial (13), as well as to evaluate the possible predictive value of this signature. Briefly, normalized gene expression estimates were downloaded from the Gene Expression Omnibus (GEO), with ID GSE117556. This cohort contained data for 928 patients, out of which 469 were treated with R-CHOP and 459 were treated with RB-CHOP. Median follow-up was 29.37 months, and median overall survival was not reached. We created a random forest model of survival following previous specifications (7, 8), and this model was exclusively trained on the group of patients treated with R-CHOP. Out-of-bag metrics were derived for patients in this subgroup, and new predictions on patients treated with RB-CHOP were made based on the results of the training set. The values of the cumulative hazard function were used to calculate the c-indexes.

## Results

Firstly, we decided to reproduce the machine learning predictions based on the expression of 17 out of 19 original genes. This was due to the fact that 2 genes (*FAM208B* and *TRAV6*) were not included in the Illumina HumanHT-12 WG-DASL V4.0 R2 expression beadchips. In the original UK population-based Haematological Malignancy Research Network database, the c-index of this signature was 0.612. In the case of the REMoDL-B trial cohort, the c-indexes were 0.619 and 0.640 for the R-CHOP and RB-CHOP treated patients, respectively. Then, we reasoned that the expression of one of the missing genes (the T-cell receptor alpha subunit variable region gene; *TRAV6*), could be substituted by the expression of the CD3 T-cell specific marker genes, namely *CD3D*, *CD3G* and *CD3E*. We observed that this strategy improved the c-index in the UK population-based Haematological Malignancy Research Network database (original c-index, 0.612; new c-index, 0.621). Hence, we performed the same modification in the REMoDL-B trial cohort, obtaining a c-index of 0.668 in the group of patients treated with R-CHOP, and a small reduction of the c-index to 0.631 in those treated with RB-CHOP.

**Figure 1:**
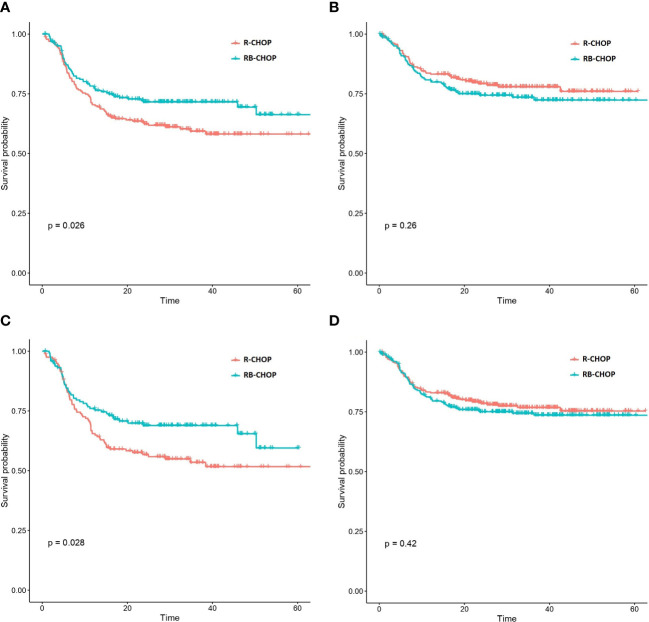
Kaplan-Meier curves representing the PFS of patients treated with RB-CHOP and R-CHOP according to their biological risk predicted by the CD3 markers & 17 gene expression signature. **(A, B)** Representation of RB-CHOP and R-CHOP curves for those patients in the higher 50% risk group **(A)** and in the lower 50%risk group **(B)**. **(C, D)** Representation of RB-CHOP and R-CHOP curves for those patients in the higher 33% risk group **(A)** and in the lower 67%risk group **(B)**.

In a second approach, we evaluated the possible predictive value of this signature in the REMoDL-B trial. With this aim, we extracted the 2-year survival probabilities from the machine learning predictions. We chose this threshold because most of the relapses and lymphoma-related deaths are known to occur during this period of time (14). Initially, we explored the possible utility of the 17-gene model by splitting the patients into 2 halves and 3 tertiles of risk (**Table 1**). No statistically significant difference in PFS was observed between patients treated with RB-CHOP and R-CHOP in either the high or the low 50% risk groups. However, a significant advantage of RB-CHOP for those patients assigned to the higher 33% of risk was identified (p-value 0.03, HR 0.66). Then, we reproduced the same procedure with the model enriched in T-cell markers (**Table 1**). In this case, we observed a significantly higher PFS with RB-CHOP for the 50% of patients at higher risk (p-value 0.03, HR 0.70; **Figure 1A**), whereas no significant differences were observed for those patients assigned to the lower 50% of risk (**Figure 1B**). This effect appeared to be even more pronounced among patients in the higher 33% of risk (p-value 0.03, HR 0.66) (**Figures 1C, D**).

**Table 1:** Results of the cox models testing for differential PFS outcomes of the different groups of patients analyzed in the text.

	17-gene model		CD3 genes + 17-gene model	
Groups	P-value	HR (95% CI)	P-value	HR (95% CI)
**50% higher risk**	0.29	0.84 (0.61-1.16)	2.94 x 10-2	0.70 (0.51-0.96)
**50% lower risk**	0.92	0.98 (0.67-1.43)	0.26	1.24 (0.85-1.82)
**33% higher risk**	3.5 x 10-2	0.66 (0.45-0.97)	2.88 x 10-2	0.66 (0.45-0.96)
**34-65% risk**	0.35	1.23 (0.79-1.92)	0.84	1.05 (0.67-1.65)
**33% lower risk**	0.97	0.99 (0.62-1.59)	0.36	1.25 (0.78-2.01)

P-values, hazard ratios (HR) and 95% confidence intervals of the HR are provided.

## Discussion

Our data indicates a role for bortezomib-containing upfront treatments in patients with DLBCL who have high-risk molecular features. According to these results, the RB-CHOP scheme achieved a 30% reduction in the risk of progression or death for the 50% of DLBCL patients at higher molecular risk, potentially expanding the effectiveness of this treatment to a wider patient population as compared with other previously defined risk groups. Furthermore, we confirmed that the inclusion of T-cell markers in the gene expression signature enriches the prognostic performance of the signature in patients treated with R-CHOP, although their importance appears to diminish in patients treated with RB-CHOP. In conclusion, the standardization and implementation of machine learning-guided molecular risk scores based on transcriptomic features should be performed in the context of clinical trials evaluating novel upfront combinations in the upfront treatment of DLBCL. Additionally, the LymForest molecular profile improves previous transcriptomic signatures for both prognostication and drug-response prediction in patients with DLBCL requiring systemic immunochemotherapy. This strategy could also be explored to enrich the results of other trials aiming to improve R-CHOP as upfront treatment in DLBCL, such as those based on polatuzumab (pola-R-CHP) (10) and those aiming to incorporate immunotherapy (bispecific antibodies or CAR-T cells) (15, 16). This is relevant because most of the new drugs in the frontline setting face a substantial difficulty to improve R-CHOP due to its high effectivity in the global population, and therefore the development of biomarkers to guide their use is of the utmost interest.

Machine learning has the potential to play an important role in the *post hoc* analysis of clinical trials by enabling more comprehensive and accurate analysis of trial data. AI-based methods can process large amounts of data and identify patterns, relationships, and insights that may not be immediately apparent through traditional statistical methods. Additionally, AI techniques can help to identify potential safety concerns, optimize dosing regimens, and identify subgroups of patients who may benefit the most from a particular treatment. For example, machine learning algorithms can be used to identify the best predictive biomarkers or clusters of patients for a particular treatment, which can inform future trial design and clinical decision making. Several studies have demonstrated the potential of AI in the *post hoc* analysis of clinical trials. For instance, a recent study by *Yan et al. (2021)* used a machine learning algorithm to predict clinical outcomes in patients with colorectal cancer treated with immunotherapy, achieving better performance than traditional statistical methods (17). Recently, newer approaches in the prediction of response to targeted drugs and drug combinations from patients treated in routine clinical practice have been presented. For example, *Kong et al. (2022)* presented an approach to predict the response to immune check-point inhibitors based on the construction of a network of genes and proteins that are known to be involved in the immune response (18). Using machine learning algorithms, they identified patterns in the network that were associated with drug response, proving that their approach can accurately predict responses in several different types of cancer. In another approach, our group explored new methods to predict risk in multiple myeloma (MM) by the integration of clinical and biochemical data with gene expression profiling. By applying machine learning algorithms, we created a 50-variable model that can predict OS with high concordance (19). The model included patient age, ISS stage, serum B2-microglobulin, first-line treatment, and the expression of 46 genes as covariates. Importantly, we found that patients treated with the best-predicted drug combination were significantly less likely to die than patients treated with other schemes, particularly those treated with a triplet combination including bortezomib, an immunomodulatory drug and dexamethasone.

Validated and transparent machine learning algorithms are essential in medical applications because they can provide accurate and reliable predictions, which can aid clinicians in making optimal decisions (20). Despite this great potential, it is important to recognize their limitations and potential biases. It is crucial to fully understand the strengths and weaknesses of each algorithm and to ensure that they are appropriately validated and transparent. This requires ongoing research and collaboration between machine learning experts and clinicians (21). In the particular context of clinical trials, *post hoc* analysis can be used to analyze data from clinical trials and determine if a drug is effective and safe (22). Machine learning can be used to identify patterns and relationships that can later be used to develop new drugs or optimize existing ones. However, *post hoc* studies also have limitations, including the possibility of data overfitting and the inability to control for confounding variables due to their retrospective nature (23). In our particular case, we retrained a previously described prognostic model in the group of patients treated with R-CHOP, because this population was the target of our predictor (6–8). Afterwards, we used these predictions to risk stratify those patients treated with RB-CHOP and compare outcomes. However, though the use of an external cohort for training the predictor could be an option, this should have the same structure (e.g., inclusion and exclusion criteria, baseline characteristics…) as the original clinical trial data. This highlights the crucial importance of external validity in clinical trials, particularly for the construction of new machine learning predictors, and the need to discuss these issues with regulatory agencies for drug approval based on such types of evidence (24). Surely, a prospective validation of the findings on a new clinical trial or in a post-authorization real world cohort would provide more reliable information. Eventually, the growing application of machine learning in clinical trials will make these *post hoc* analysis more relevant, and regulators should pursue the development of good clinical practices to ensure a reliable and fair application (25, 26). This includes using appropriate statistical methods, validating the model on multiple datasets, and being transparent about its possible limitations. Another issue of relevance for the application of this technology relies on the need for genomic standardization, which should be pursued in order to lead to reliable results for patient care. Standardization of genomic tests involves ensuring that the tests are performed in a consistent and reliable manner across different laboratories, using well-defined protocols, standardized testing platforms and quality control measures (27). This will help to ensure that the results of the tests are accurate and can be compared across different settings and over time.

In conclusion, we present an evaluation of LymForest-25 machine-learning-based gene expression profile to risk stratify patients and predict treatment responses in patients with DLBCL within the REMoDL-B trial. The results suggest a role for bortezomib-containing upfront treatments in molecular high-risk patients. The standardization and implementation of machine learning-guided molecular risk scores based on transcriptomic features should be pursued in the context of clinical trials evaluating novel upfront combinations in the upfront treatment of DLBCL.

## Data availability statement

The datasets presented in this study can be found in online repositories. The names of the repository/repositories and accession number(s) can be found in the article/supplementary material.

## Author contributions

AMO had the idea, designed the paper, analyzed the data and wrote the paper. JADA, MCL, APR, LBP, MSGP, MMPE, MFFR and JLBL reviewed the results, suggested modifications and approved the final publication. All authors contributed to the article and approved the submitted version.
